# Reassortant High Pathogenicity Avian Influenza A(H5N1) Viruses During the Reemergence in Uruguay Suggest Increasing Genetic Diversity in South America

**DOI:** 10.3390/v18050558

**Published:** 2026-05-14

**Authors:** Ana Marandino, Gonzalo Tomás, Yanina Panzera, Valeria Uriarte, Virginia Russi, Ramiro Pérez, Lucía Bassetti, Raúl Negro, Sirley Rodríguez, Ruben Pérez

**Affiliations:** 1 Sección Genética Evolutiva, Departamento de Biología Animal, Instituto de Biología, Facultad de Ciencias, Universidad de la República, Iguá 4225, Montevideo 11400, Uruguay; amarandino@fcien.edu.uy (A.M.); gtomas@fcien.edu.uy (G.T.); ypanzera@fcien.edu.uy (Y.P.); 2 Dirección Nacional de Biodiversidad y Servicios Ecosistémicos (DINABISE), Ministerio de Ambiente, Juncal 1385, Montevideo 11100, Uruguay; valeria.uriarte@ambiente.gub.uy; 3 División de Sanidad Animal, Dirección General de Servicios Ganaderos, Ministerio de Ganadería, Agricultura y Pesca, Ruta 8 “Brigadier Gral. Juan A. Lavalleja” Km 17,000, Montevideo 12100, Uruguay; vrussi@mgap.gub.uy; 4 Departamento de Virología, División de Laboratorios Veterinarios “Miguel C. Rubino”, Dirección General de Servicios Ganaderos, Ministerio de Ganadería, Agricultura y Pesca, Ruta 8 “Brigadier Gral. Juan A. Lavalleja” Km 17,000, Montevideo 12100, Uruguay; raperez@mgap.gub.uy (R.P.); lbassetti@mgap.gub.uy (L.B.); rnegro@mgap.gub.uy (R.N.); srodriguez@mgap.gub.uy (S.R.)

**Keywords:** H5N1, avian influenza, reassortment, phylogenetics, genomic surveillance, South America, RT-qPCR, viral evolution

## Abstract

Highly pathogenic avian influenza (HPAI) H5N1 viruses of the goose/Guangdong (Gs/GD) lineage have driven a global panzootic since 2020, with clade 2.3.4.4b establishing sustained transmission in wild birds. In South America, early outbreaks were largely associated with the North American-derived B3.2 genotype, which showed limited diversification after its introduction. Here, we report the genomic characterization of eight H5N1 viruses detected in Uruguay during the reemergence of avian influenza in February–March 2026. Complete genomes were obtained from wild birds exhibiting neurological signs, predominantly *Coscoroba coscoroba*. All viruses belong to clade 2.3.4.4b but exhibit a reassortant genomic constellation distinct from B3.2. The HA, NA, and MP segments retain the Eurasian backbone, whereas internal genes derive from both South American and North American low-pathogenicity avian influenza lineages. PB2 variation distinguishes two closely related viral groups differing in PB2 origin, whereas the remaining genomic segments retain a shared background. Sequence variation in the neuraminidase gene reduced the sensitivity of a widely used N1-specific RT-qPCR assay, highlighting limitations of existing diagnostic tools during viral evolution. These findings confirm the presence of reassortant H5N1 viruses in Uruguay and, together with recent reports from Argentina and Brazil, support an emerging pattern of genomic diversification in southern South America.

## 1. Introduction

Avian influenza viruses (AIVs) belong to the genus *Alphainfluenzavirus* in the family Orthomyxoviridae and are important pathogens of both wildlife and domestic poultry [1,2]. Wild aquatic birds, particularly species within the orders Anseriformes and Charadriiformes, constitute the primary natural reservoirs in which AIVs typically circulate as low-pathogenicity avian influenza (LPAI) viruses [2,3]. Low-pathogenicity avian influenza viruses can evolve into highly pathogenic forms by acquiring a polybasic cleavage site in the hemagglutinin (HA) protein. This process may occur primarily in viruses of the H5 and H7 subtypes when circulating in populations of galliform poultry. However, the conditions and prerequisites for such a mutation remain unclear. Genetic diversity in influenza A viruses arises through both mutation and reassortment: mutation is generated by the error-prone viral RNA polymerase, whereas the segmented genome facilitates reassortment during coinfection with genetically distinct viruses [4,5].

Over the past decade, HPAI H5 viruses of the goose/Guangdong (Gs/GD) lineage, particularly those belonging to clade 2.3.4.4b, have driven a global panzootic affecting wild birds, poultry, and several mammalian species [6]. These viruses have spread across multiple continents, establishing transmission cycles in diverse ecological settings [7,8]. Clade 2.3.4.4b viruses were first detected in North America in late 2021 [8,9,10], most likely following introduction via migratory birds, and subsequently expanded southward into Central and South America [11,12,13,14].

During the early phase of the South American epizootic, the viral population was largely dominated by a single genotype, B3.2, a reassortant constellation combining Eurasian-derived surface genes (HA, NA, and MP) with internal segments originating from North American LPAI viruses. This genotype showed limited genomic diversification during its initial spread across the continent, consistent with a scenario of recent introduction followed by regional dissemination [12,15,16,17,18,19].

More recently, however, genomic studies from Argentina and Brazil have reported the emergence of reassortant H5N1 viruses incorporating gene segments from South American LPAI lineages, indicating increased interaction between highly pathogenic viruses and local avian influenza reservoirs [20,21,22]. We hypothesize that the evolutionary dynamics of H5N1 in South America are transitioning from an initial phase of relative genetic homogeneity toward a more complex scenario characterized by increasing reassortment and genomic diversification.

Uruguay is a key geographic interface among southern South American countries, linking inland wetlands, coastal ecosystems, and migratory flyways. The country reported its first H5N1 outbreaks caused by the B3.2 genotype in February 2023, which affected wild birds, backyard poultry, and marine mammals [14]. The last detection before the 2026 outbreak was reported in October 2023, when a royal tern (*Thalasseus maximus*) was found dead on Isla de Flores, an offshore island between the departments of Montevideo and Canelones [16].

After approximately 28 months without reported H5N1 detections in Uruguay, new cases were identified in February 2026, providing an opportunity to investigate the genomic characteristics of currently circulating viruses in the region.

In this study, we generated and analyzed complete genomes of H5N1 viruses detected in Uruguay during the 2026 outbreak to characterize their genomic constellations, determine their relationship with previously described South American viruses, and assess the roles of reassortment and local diversification in shaping the current evolutionary landscape of H5N1 in southern South America.

## 2. Materials and Methods

### 2.1. Sample Collection

**Figure 1:**
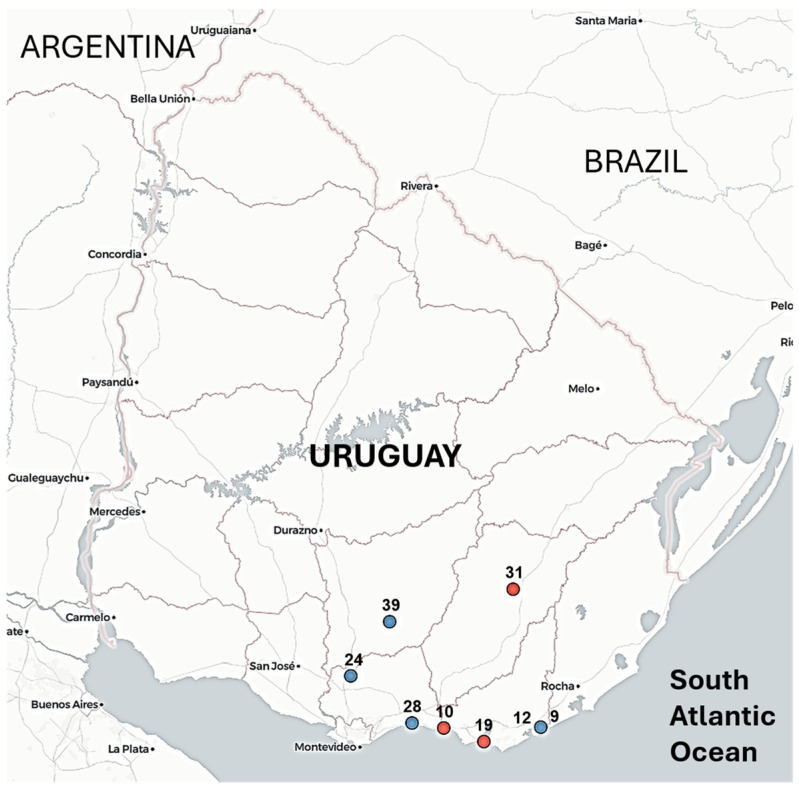
Geographic distribution of H5N1 viruses analyzed in this study. Sampling locations of H5N1-positive wild birds included in this study. Points are colored by genotype: SA-1a (red) and SA-1b (blue). Sample 19 corresponds to *Cygnus melancoryphus* (black-necked swan), whereas all other samples correspond to *Coscoroba coscoroba* (coscoroba swan, a goose-like swan). Locations represent cases from which complete or near-complete genomes were obtained.

Wild birds found dead or showing neurological signs were collected between February and March 2026 as part of coordinated surveillance by Uruguay’s animal health services (the Ministry of Environment and the Ministry of Livestock, Agriculture, and Fisheries). Most birds in this study were *Coscoroba coscoroba* (Coscoroba swan), a species endemic to southern South America, characterized by its white plumage and relatively small size compared to other swans; one additional sample was obtained from *Cygnus melancoryphus* (black-necked swan). Sampling sites included wetlands and coastal areas in southern Uruguay (Figure 1). During the surveillance period, 32 additional wild birds with compatible clinical signs or found dead tested negative for avian influenza virus. No cases were detected in backyard or commercial poultry during the 2026 outbreak, unlike the 2023 epidemic in Uruguay.

Cloacal and brain tissue swabs were collected from each animal and placed in sterile tubes containing Dulbecco’s Modified Eagle Medium (DMEM; Sigma-Aldrich, St. Louis, MO, USA) supplemented with antibiotics and antifungal agents. Samples were refrigerated during transport and processed upon arrival at the laboratory.

### 2.2. Detection and Typing of Avian Influenza Virus

Viral RNA was extracted using the TACO™ mini Automatic Nucleic Acid Extraction System (GeneReach Biotechnology, Taichung, Taiwan) following the manufacturer’s instructions. Detection and characterization of avian influenza virus were performed using real-time reverse transcription PCR (RT-qPCR) targeting three genomic regions: the matrix (M) gene, the hemagglutinin (H5) gene, and the neuraminidase (N1) gene.

Screening for influenza A virus was conducted using the ID Gene™ Influenza A Duplex assay (Innovative Diagnostics, Grabels, France), which targets the conserved matrix (M) gene. Subtyping of H5 viruses (clade 2.3.4.4) was performed using the validated RT-qPCR protocol NVSL-WI-1732.02 from the National Veterinary Services Laboratories (NVSL) of the United States Department of Agriculture (USDA).

Detection of the neuraminidase N1 subtype was performed using the RT-qPCR assay included in the official NVSL diagnostic workflow (NVSL-WI-1768, Revision 01), which targets the Eurasian-lineage N1 gene associated with clade 2.3.4.4b H5N1 viruses. The assay used the following oligonucleotides: forward primer 2022_N1+192 (5′-GACGTATGTCAACATCAGCAATAC-3′), probe 2022_N1-250 ([FAM]-TTACCGAAG/ZEN/TAACAGCCTGCTCAGC-[NFQ]), and reverse primer 2022_N1-290 (5′-CACCCACTAATAGGGCAAAGA-3′).

To assess assay performance, seven samples from the 2023 outbreak were used as positive controls and processed under the same diagnostic conditions.

All amplification reactions were performed on a MIC-4 real-time thermal cycler (BioMolecular Systems, Upper Coomera, QLD, Australia) under validated diagnostic conditions using hydrolysis probe chemistry. Cycle threshold (Ct) values were obtained for each target (M, H5, and N1) and used to assess viral detection and subtype characterization. Results were interpreted according to established diagnostic criteria. Samples with positive amplification were selected for whole-genome sequencing.

### 2.3. cDNA Synthesis and Amplification of Viral Genomes

Complementary DNA (cDNA) was synthesized with SuperScript™ II Reverse Transcriptase (Invitrogen, Carlsbad, CA, USA) according to the manufacturer’s protocol.

Amplification of the eight genomic segments of the influenza A virus was performed using a multisegment RT-PCR approach with universal influenza primers and Q5^®^ High-Fidelity DNA Polymerase (New England Biolabs, Ipswich, MA, USA), as previously described [23]. This method enables the simultaneous amplification of the PB2, PB1, PA, HA, NP, NA, M, and NS segments.

PCR products were visualized by agarose gel electrophoresis and purified before library preparation.

### 2.4. Library Preparation and Next-Generation Sequencing

Sequencing libraries were prepared with the Illumina DNA Prep Kit (Illumina, San Diego, CA, USA) according to the manufacturer’s instructions. Libraries were purified with AMPure XP magnetic beads (Beckman Coulter, Brea, CA, USA) and quantified with the Qubit™ dsDNA High Sensitivity Assay Kit (Thermo Fisher Scientific, Waltham, MA, USA).

Library quality and fragment-size distribution were assessed using a Fragment Analyzer™ system (Agilent Technologies, Santa Clara, CA, USA).

Sequencing was performed on an Illumina MiSeq i100 Plus platform using 2 × 150 bp paired-end sequencing.

### 2.5. Genome Assembly and Annotation

Raw sequencing reads were imported into Geneious Prime (version 2026.0.2; Biomatters Ltd., Auckland, New Zealand) for quality control, trimming, and assembly. Low-quality bases and adapter sequences were trimmed before assembly [24].

Consensus sequences were generated using a reference-guided approach based on representative H5N1 genomes. Assemblies were manually inspected to verify coverage and resolve ambiguous positions.

Genome annotation and segment identification were performed using sequence similarity to reference influenza A virus genomes available in public databases, including GISAID and GenBank.

### 2.6. Nucleotide Divergence Analysis

Coding sequences were aligned for each genomic segment, and pairwise nucleotide divergence was computed between predefined groups.

Total nucleotide divergence was expressed as a percentage using the formula (d/L) × 100, where d is the mean number of nucleotide differences, and L is the segment length.

### 2.7. Phylogenetic Analysis

Representative influenza A virus sequences from South America, North America, and the global H5N1 clade 2.3.4.4b datasets were retrieved from public databases.

Multiple sequence alignments were performed with MAFFT v7 [25].

Phylogenetic trees were inferred with FastTree v2.1 [26] under the generalized time-reversible (GTR) model with gamma-distributed rate variation across sites. Trees were visualized and annotated to determine the evolutionary placement of Uruguayan viruses relative to previously described lineages.

Segment lineage assignments were determined using a combination of phylogenetic clustering, GenoFLU classification, exploratory analysis with the ggFlu web server (https://www.ggflu.org/, accessed on 10 May 2026), and regional genomic context [10]. Reassortment patterns were inferred from incongruence among segment phylogenies.

## 3. Results

### 3.1. Description of the Outbreak

The reemergence of avian influenza in Uruguay was first detected on 18 February 2026 in Laguna Garzón, a coastal lagoon between the Maldonado and Rocha departments (Figure 1, Table 1). The index case was *Coscoroba coscoroba*, a South American swan-like species with a goose-like appearance.

After the initial detection, seven additional positive cases were identified across southern Uruguay (Figure 1, Table 1), indicating spatial spread beyond the index site. Seven cases involved Coscoroba swans, whereas a single case was detected in *Cygnus melancoryphus* (black-necked swan) approximately 50 km from the initial outbreak focus.

Clinical presentation was consistently marked by neurological signs, including ataxia, abnormal posture, inability to fly, and reduced responsiveness, often preceding death (Table 1).

### 3.2. Genome Recovery and General Features

Eight H5N1 genomes were obtained from samples collected in February–March 2026. Six genomes contained the complete set of eight canonical influenza A segments (PB2, PB1, PA, HA, NP, NA, MP, and NS) and showed high coverage (>100×) across coding regions. Two genomes had partial PB1 sequences, but the remaining seven segments were complete.

The complete coding regions totaled 13,136 nucleotides per genome. All consensus sequences were generated and submitted to GenBank (Table 1).

BLAST analysis of individual genomic segments using NCBI BLASTn v2.17.0 indicated that the closest related sequences mainly correspond to viruses reported in Argentina and Brazil in 2025, suggesting a regional evolutionary link across these outbreaks.

### 3.3. Segment-Specific Phylogenetic Relationships and Genomic Constellation

**Figure 2:**
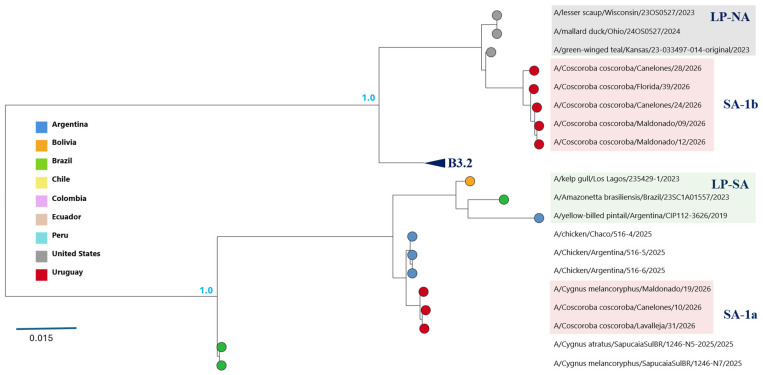
Phylogenetic structure of the PB2 segment. Maximum-likelihood phylogenetic tree of the PB2 segment showing the distribution of South American H5N1 viruses. Uruguayan viruses detected in 2026 form two groups: SA-1a and SA-1b (red shaded boxes). Tip colors indicate the country of origin. The SA-1b group forms a well-supported monophyletic clade within a North American low-pathogenicity avian influenza lineage (am2.1), which also includes the B3.2 genotype (collapsed in the figure). In contrast, SA-1a clusters separately within South American low-pathogenicity lineages (LP-SA), consistent with distinct evolutionary histories involving reassortment. Branch lengths are proportional to the number of substitutions per site.

**Figure 3:**
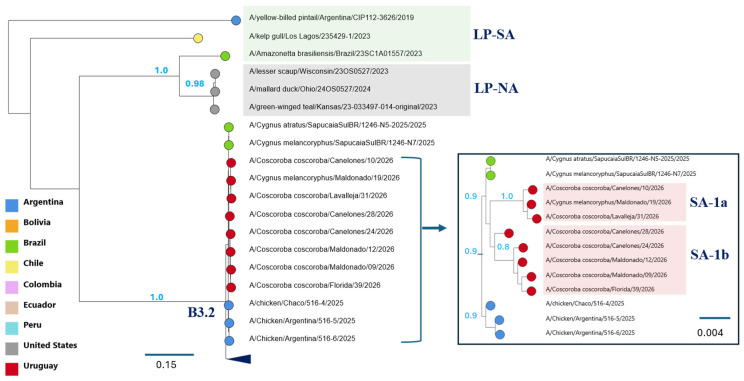
Phylogenetic structure of the HA segment (segment 4). Maximum-likelihood phylogenetic tree of the HA segment showing that Uruguayan viruses cluster within the Eurasian H5N1 clade 2.3.4.4b backbone and are clearly differentiated from South American (LP-SA) and North American (LP-NA) low-pathogenicity avian influenza lineages. Tip colors indicate the country of origin. Due to the low number of substitutions per site, the clade containing Uruguayan viruses is expanded (boxed) to improve visualization. Within this clade, a weak phylogenetic structure is observed, with sequences from SA-1a and SA-1b tending to form separate subclusters, although genetic divergence is limited and supported by few nucleotide substitutions. Branch lengths are proportional to the number of substitutions per site.

Phylogenetic analyses revealed segment-specific evolutionary relationships among the Uruguayan viruses, with distinct clustering patterns across the viral genome (Figure 2 and Figure 3; Supplementary Figure S2; Table 2). To characterize these patterns, each genomic segment was analyzed separately.

PB2 (Segment 1)

The PB2 segment was divided into two clearly differentiated lineages (Figure 2). Three viruses (10, 19, and 31) clustered with South American low-pathogenicity avian influenza (LPAI) lineages (LP-SA), designated here as the SA-1a group. In contrast, five viruses (9, 12, 24, 28, and 39) grouped with North American LPAI lineages within the am2.1 clade and were designated as the SA-1b group.

The nucleotide divergence between these two PB2 groups was high (17.4%) and included single-nucleotide substitutions, with no insertions or deletions detected. Relative to the Uruguayan virus detected in 2023, PB2 divergence was higher in SA-1a (17.8%) than in SA-1b (5.1%) (Supplementary Figure S1).

These results identify two distinct groups of viruses based on PB2 phylogenetic relationships.

PB1 (Segment 2), PA (Segment 3), and NS (Segment 8)

In contrast to PB2, the PB1, PA, and NS segments were highly conserved across all Uruguayan viruses and consistently clustered with South American low-pathogenicity avian influenza (LPAI) lineages (LP-SA). These segments were clearly distinct from those of the Uruguayan strain from the 2023 outbreak, with nucleotide divergence ranging from 10.1% to 15.2% (Supplementary Figure S1).

A subtle phylogenetic separation between the SA-1a and SA-1b groups was also observed in these segments (Supplementary Figure S2).

NP (Segment 5)

The NP segment clustered with North American LPAI viruses and was classified within the am1.1 lineage (Supplementary Figures S1 and S2E). This segment differed from the am1.4.1 lineage associated with the B3.2 genotype detected in Uruguay in 2023, with an average nucleotide divergence of approximately 4.4%. Phylogenetic analysis showed that, although all NP segments derive from North American LPAI lineages, the NP segment in the 2026 viruses does not share a recent common ancestor with the B3.2 lineage, suggesting independent acquisition events.

Minor phylogenetic differences between the SA-1a and SA-1b groups were also observed in this segment.

HA (Segment 4), NA (Segment 6), and MP (Segment 7)

The HA, NA, and MP segments were highly conserved across all samples and clustered within the Eurasian H5N1 clade 2.3.4.4b backbone (ea1 lineage) (Figure 3 and Supplementary Figure S2D,F,G). All viruses contained the characteristic polybasic cleavage site motif (PLREKR-RKR/G), consistent with highly pathogenic phenotypes.

The SA-1a and SA-1b viruses clustered within a broader clade that also included strains from Argentina and Brazil collected in 2025. Within this clade, the internal phylogenetic structure was weak, with sequences from each group tending to form distinct subclades. The low nucleotide divergence (<1%) within this clade likely contributes to the limited resolution of this substructure (Supplementary Figures S1 and S2D–G).

### 3.4. Genomic Classification of Uruguayan Viruses

Based on PB2 phylogenetic clustering, two genomic groups were identified among the Uruguayan viruses detected in 2026. The SA-1a group carries a PB2 segment derived from South American low-pathogenicity avian influenza (LPAI) lineages (LP-SA), whereas the SA-1b group contains a PB2 segment of North American LPAI origin (Figure 2, Table 2). In contrast, the remaining seven genomic segments showed limited phylogenetic differentiation between groups, consistent with a largely shared genomic background (Supplementary Figure S2).

The SA-1a group shares a genomic constellation with that of reassortant viruses reported in Brazil and Argentina in 2025 (Table 2). Segment lineage assignments followed the GenoFLU classification when available, which identified the PB2 segments of SA-1b viruses as am2.1 and the NP segments of all viruses as am1.1. PB1, PA, and NS segments were assigned to LP-SA lineages based on phylogenetic analysis because GenoFLU did not classify them.

Exploratory analysis using the ggFlu platform showed partial agreement with these results. In SA-1a viruses, PB2 and NS were classified as South American lineages, whereas PB1 was assigned to a North American lineage. In addition, ggFlu assigned SA-1a viruses to the minor genotype K.8-1, while SA-1b viruses could not be assigned to a defined genotype.

### 3.5. Sequence Variation in the NA Segment and Impact on N1 RT-qPCR Detection

Despite high viral loads detected by M- and H5-specific RT-qPCR assays, the N1-specific assay showed reduced sensitivity, yielding Ct values above 35 or failing to produce detectable amplification signals, indicating that N1 detection was at or beyond the assay’s limit of detection (Table 3). Consistent with this, reanalysis of seven positive control samples from the 2023 outbreak under the same diagnostic conditions yielded Ct values ranging from 23.1 to 32.4, confirming successful amplification, whereas samples from the 2026 outbreak remained undetected.

**Figure 4:**
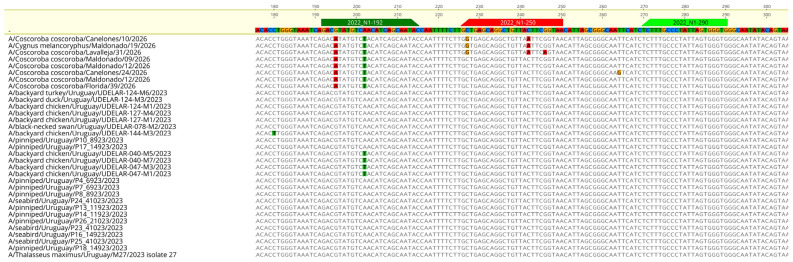
Sequence variation in the N1 RT-qPCR target region. Multiple sequence alignment of the neuraminidase (NA) gene region (N1 subtype) targeted by the RT-qPCR assay used during the 2023 and 2026 outbreaks. The binding sites of the forward primer, reverse primer, and probe are indicated. Uruguayan viruses from the 2026 outbreak show two conserved single-nucleotide polymorphisms (SNPs) within the forward primer-binding region, whereas viruses from the 2023 outbreak do not show mismatches in this region. No mismatches were detected in the reverse primer-binding site in any of the analyzed sequences. These mismatches likely contribute to the reduced sensitivity and elevated Ct values observed for the N1 RT-qPCR assay in 2026 samples (see Table 3). In contrast, substitutions in the probe-binding region were only observed in a subset of 2026 viruses (SA-1a group).

Sequence analysis of the NA gene revealed mismatches in both primer- and probe-binding regions. Two single-nucleotide polymorphisms (SNPs) were consistently identified in the forward primer-binding site across all analyzed viruses, whereas no mismatches were detected in the reverse primer-binding site. In contrast, substitutions in the probe-binding region were limited to the SA-1a genotype, with two SNPs observed in samples 10 and 19 and three SNPs in sample 31 (Figure 4). Since the mismatches in the forward primer were conserved across all analyzed viruses, these sequence variations may have contributed to the diminished sensitivity observed in the N1 RT-qPCR assay.

## 4. Discussion

The initial spread of H5N1 in South America was largely dominated by the GenoFlu-defined B3.2 genotype, which combined Eurasian-derived surface genes with internal segments of North American low-pathogenicity avian influenza (LPAI) origin (Table 2). This genotype showed limited diversification during its early expansion across the continent, consistent with a recent introduction followed by rapid regional dissemination [15,16,18,19,27]. In contrast, the viruses detected during the 2026 reemergence in Uruguay belong to a more diverse reassortant viral population circulating in South America, consistent with recent reports from Argentina and southern Brazil [20,21,22]. These viruses share a largely conserved genomic backbone across most segments composed of Eurasian-derived surface genes (HA, NA, and MP) and internal segments derived from both South American and North American LPAI lineages (Table 2).

One of the key findings of this study is that genomic differentiation among the Uruguayan viruses is primarily associated with the evolutionary origin of the PB2 segment. Phylogenetic analyses identified two well-defined groups (SA-1a and SA-1b) based on PB2 clustering, corresponding to South American and North American LPAI-derived lineages, respectively (Figure 2). By comparison, the other genomic segments did not show the same degree of phylogenetic separation.

Together, these results are consistent with a model in which differentiation between SA-1a and SA-1b is primarily driven by differences in the evolutionary origin of the PB2 segment, while the remaining genomic segments retain a largely shared lineage background. Although PB2 distinguishes the two groups, the available data do not allow the temporal sequence of events leading to this pattern to be resolved. In particular, it remains unclear whether PB2 reassortment occurred before the divergence observed in the remaining segments, or whether these viruses arose from already differentiated viral populations that subsequently acquired distinct PB2 segments. Regardless of the underlying scenario, SA-1a and SA-1b are best interpreted as closely related reassortant groups that differ primarily in PB2 origin while sharing a largely conserved genomic constellation.

The characteristics of SA-1a and SA-1b also highlight the challenges of applying existing genotype classification frameworks to South American H5N1 viruses. Phylogenetic analysis clearly supports differentiation by PB2 origin, but lineage assignments using GenoFLU were only partially resolved, leaving several internal segments unclassified and requiring phylogenetic interpretation. Similarly, an exploratory analysis using the new ggFlu platform showed partial concordance, successfully assigning SA-1a viruses to a defined minor genotype K.8-1 but failing to classify SA-1b. These discrepancies likely reflect limited representation of South American LPAI lineages and still-sparse sampling of recently emerged H5N1 reassortant viruses, suggesting that genotype definitions need further refinement.

A notable finding of this study is the reduced performance of the N1-specific RT-qPCR assay, despite high viral loads detected by H5-targeted assays (Table 3). Sequence analysis revealed mismatches in primer- and probe-binding regions. These observations suggest that NA sequence variation may have contributed to reduced assay sensitivity. Importantly, this assay had been successfully used during the 2023 outbreak in Uruguay and in previous regional surveillance efforts, supporting the interpretation that the reduced sensitivity observed in 2026 is likely strain-associated rather than indicative of general assay failure. However, additional factors, such as differences in viral RNA concentration across genomic targets, sample quality, tissue-associated PCR inhibitors, or RNA degradation, may also have influenced assay performance, as previously described for influenza A virus molecular diagnostic assays [28,29]. While further validation is needed to determine the relative contributions of these factors, the findings underscore that viral evolution can affect molecular diagnostics as viral populations diversify and reassort, and that regular evaluation of molecular assays is important for accurate detection and subtyping.

From an epidemiological perspective, the 2026 detections represent the reemergence of H5N1 in Uruguay after a period without reported cases in 2024–2025. This temporal gap may reflect either a true interruption in detectable viral circulation or low-level viral persistence that remained undetected by passive surveillance systems, which primarily rely on identifying symptomatic or dead birds. The predominance of cases in *Coscoroba coscoroba*, with only a single detection in *Cygnus melancoryphus*, suggests a shift in the host species affected compared with earlier outbreaks. Although *Coscoroba coscoroba* is not traditionally considered a primary host in H5N1 epidemiology, its widespread distribution across southern South American wetlands and its ecological interactions with other waterfowl species may facilitate viral transmission. Interestingly, as observed in this and previous outbreaks, initial detections often involve swan species, including *Cygnus* spp., which have been reported as early indicators of H5N1 presence in multiple regions worldwide [30]. This likely reflects their high susceptibility to highly pathogenic avian influenza viruses, combined with their large body size and visibility, which increase the likelihood of detection during passive surveillance. In this context, swans may serve as effective sentinel species for viral circulation, particularly in systems that rely on detecting symptomatic or dead birds. These observations are consistent with a scenario in which local ecological factors, including host community composition, species-specific susceptibility, and detectability, influence outbreak dynamics.

Regional comparisons further support the interpretation that South America is transitioning toward greater genomic diversification of H5N1 viruses. In Argentina, recent outbreaks have been linked to reassortant viruses carrying segments from Eurasian, North American, and South American lineages, while studies from Brazil have documented multiple introductions and intra-epidemic reassortment events [20,21,31]. The genomic constellation observed in Uruguay aligns with these patterns, suggesting that similar evolutionary processes are occurring across the southern South American countries. Rather than isolated events, the emergence of reassortant viruses in Uruguay, Argentina, and Brazil likely reflects a shared regional dynamic driven by interactions between HPAI viruses and endemic LPAI reservoirs.

An important implication of this study is that a single genotype may no longer dominate the South American H5N1 epidemic, as it did during the initial phase of introduction. Instead, the increasing detection of reassortant variants and the coexistence of closely related yet genetically distinct viral groups suggest a shift toward a more complex evolutionary landscape [32,33]. In this context, the identification of genotype groups such as SA-1a and SA-1b should be interpreted as reflecting early stages of genomic differentiation within a shared viral population, rather than fully independent evolutionary lineages. This distinction is particularly relevant for understanding the tempo and mode of influenza virus evolution in newly colonized regions [5].

Finally, these findings underscore the importance of sustained genomic surveillance in South America. The detection of gene segments from both North and South American LPAI lineages highlights local viral diversity as a source of reassortment. However, the availability of genomic data on LPAI viruses in the region remains limited, affecting the ability to reconstruct reassortment pathways and fully identify ancestral sources [27,34,35,36,37,38]. Expanding surveillance to include a broader range of wild bird species and ecological settings will be critical to improving our understanding of viral evolution and anticipating future changes in the epidemiology of H5N1 [39].

## 5. Conclusions

This study documents the reemergence of H5N1 in Uruguay and supports recent observations from Brazil and Argentina suggesting increasing genomic diversity among viruses in southern South America. The identification of two closely related viral groups differing in PB2 origin, together with evidence of reassortment between South and North American lineages, highlights the dynamic evolutionary landscape of H5N1 in the region. In addition, sequence variation in primer- and probe-binding regions may have contributed to the observed reduced sensitivity of the N1 RT-qPCR assay, underscoring the need to periodically evaluate diagnostic tools as viral populations evolve. Overall, these findings reinforce the value of sustained genomic surveillance to monitor viral evolution and to support epidemiological surveillance and control strategies.

## Figures and Tables

**Table 1:** Samples used in the study.

Strain	Origin	Host Species	Date	Clinical Signs	Accession Numbers
9	Maldonado	*Coscoroba coscoroba*	18 February 2026	Neurological signs: inability to stand or fly; neck weakness	PZ295020–PZ295027
10	Canelones	*Coscoroba coscoroba*	22 February 2026	Neurological signs: lethargy; reduced escape behavior	PZ294980–PZ294987
12	Maldonado	*Coscoroba coscoroba*	23 February 2026	Neurological signs: severe condition; euthanized	PZ295012–PZ295019
19	Maldonado	*Cygnus melancoryphus*	26 February 2026	Neurological signs	PZ295028–PZ295035
24	Canelones	*Coscoroba coscoroba*	27 February 2026	Neurological signs: difficulty standing; abnormal pecking behavior	PZ294988–PZ294995
28	Canelones	*Coscoroba coscoroba*	1 March 2026	Neurological signs	PZ294973–PZ294979
31	Lavalleja	*Coscoroba coscoroba*	3 March 2026	Neurological signs	PZ295004–PZ295011
39	Florida	*Coscoroba coscoroba*	5 March 2026	Neurological signs: dropped neck; inability to fly; depression; head-down posture	PZ294996–PZ295003

Clinical signs were recorded during field surveillance. Neurological signs included impaired coordination, abnormal posture, and reduced responsiveness. “Reduced escape behavior” indicates marked lethargy, and “dropped neck” refers to flaccid cervical posture. Euthanasia was performed in cases of severe clinical deterioration.

**Table 2:** Genomic constellations of H5N1 viruses detected in Uruguay in 2026.

Strains	PB2	PB1	PA	HA	NP	NA	MP	NS	Genotype Group
10, 19 and 31	LP-SA	LP-SA	LP-SA	ea1	am1.1	ea1	ea1	LP-SA	SA-1a
9, 12, 24, 28 and 39	am2.1	LP-SA	LP-SA	ea1	am1.1	ea1	ea1	LP-SA	SA-1b
	am2.1	am1.X	ea1	ea1	am1.4.1	ea1	ea1	am1.1	B3.2

Segment classification was based on phylogenetic analysis. All viruses shared a Eurasian-origin HA, NA, and MP backbone (ea1), while internal segments showed reassortment with South American (LP-SA) and North American (am) LPAI lineages. Two genotype groups (SA-1a and SA-1b) were identified, which differed in the origin of the PB2 gene. The GenoFlu-defined B3.2 genotype is included for comparison. Abbreviations: LPAI, low-pathogenicity avian influenza; LP-SA, South American lineage; am, North American lineage; ea, Eurasian lineage.

**Table 3:** Ct values obtained by RT-qPCR targeting the influenza A virus matrix (M), hemagglutinin (H) 5, and neuraminidase (N) 1 genes in samples collected in Uruguay during 2026.

Strain	Sample (Swab)	M Ct	N1 Ct	H5 Ct
9	Tracheal	24.18	ND	NA
9	Brain	16.91	NA	16.47
10	Brain	17.44	35.69	19.67
12	Cloacal	22.35	ND	NA
19	Tracheal/cloacal	23.07	ND	24.57
24	Brain	23.81	ND	25.32
28	Brain	25.20	ND	28.87
31	Oropharyngeal	31.35	ND	NA
39	Brain	18.36	36.44	NA

Cycle threshold (Ct) values are shown for each target and sample type. ND, not detected (no amplification signal observed or Ct value above the limit of detection); NA, not available (assay not performed or result is unavailable).

## Data Availability

All sequence data generated in this study were submitted to GenBank. Accession numbers are listed in Table 1.

## Supplementary Materials

The following supporting information can be downloaded at: https://www.mdpi.com/article/10.3390/v18050558/s1, Figure S1: Segment-specific nucleotide divergence among Uruguayan H5N1 viruses; Figure S2: (A–H) Phylogenetic structure of all eight gene segments of H5N1 viruses detected in South America.
