# Supplementary material for: Reassortant High Pathogenicity Avian Influenza A(H5N1) Viruses During the Reemergence in Uruguay Suggest Increasing Genetic Diversity in South America
Source: Viruses. 2026 May 14;18(5):558. doi: 10.3390/v18050558 (PMC13211659; doi:10.3390/v18050558)

Supplementary Figure S1. Segment-specific nucleotide divergence among Uruguayan H5N1 viruses.

Heatmap showing the percentage nucleotide divergence between groups of viruses detected in Uruguay in 2026 (1a and 1b) and viruses detected in 2023 (genotype B3.2), as well as pairwise comparisons between groups 1a and 1b. Divergence values were calculated as (dN + dS) / l × 100, where l represents the mean number of nucleotides corresponding to the segment length.

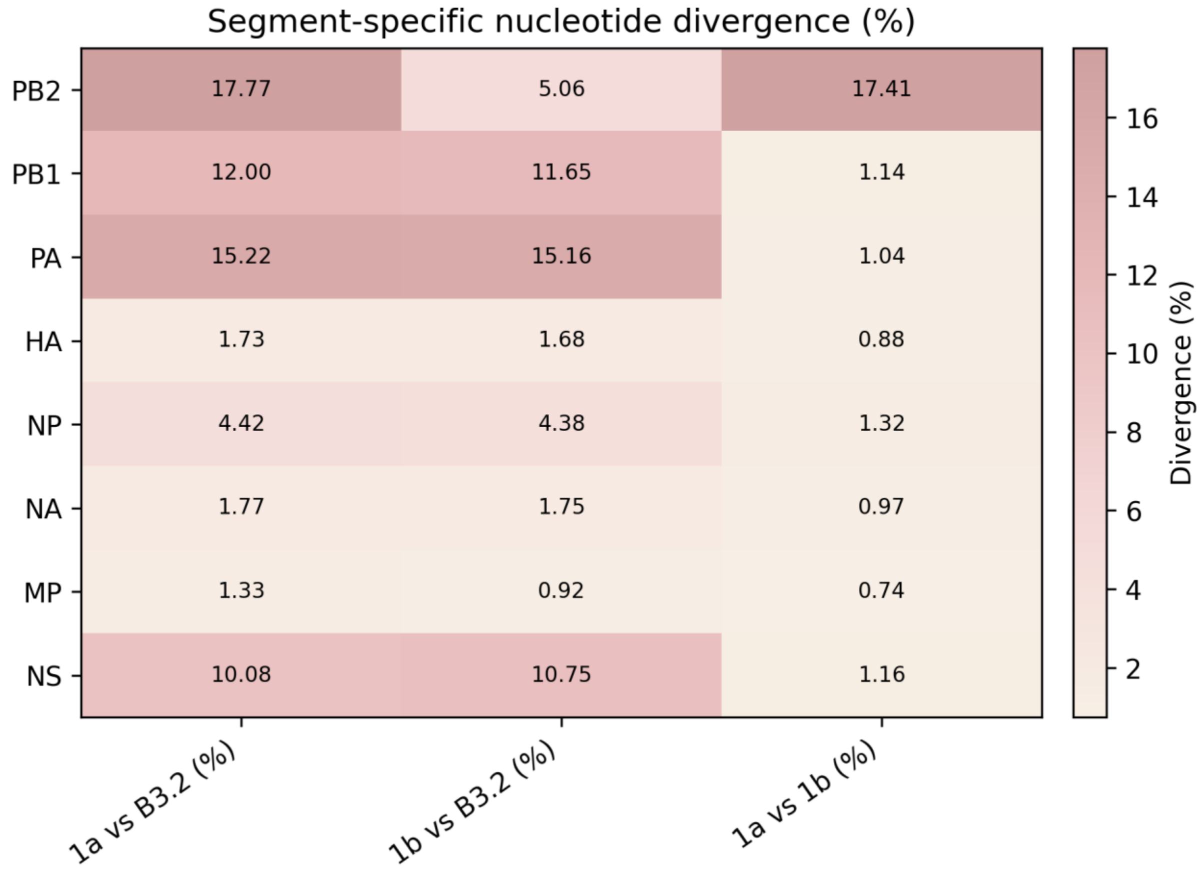

Supplement: Supplementary file 1 [file viruses-18-00558-s001.zip › Supplementary Figure S1.pdf]
