# Supplementary material for: Reassortant High Pathogenicity Avian Influenza A(H5N1) Viruses During the Reemergence in Uruguay Suggest Increasing Genetic Diversity in South America
Source: Viruses. 2026 May 14;18(5):558. doi: 10.3390/v18050558 (PMC13211659; doi:10.3390/v18050558)

Figure S2. (A–H) Phylogenetic structure of all eight gene segments of H5N1 viruses detected in South America.
Maximum-likelihood phylogenetic trees for each genomic segment: (A) PB2, (B) PB1, (C) PA, (D) HA, (E) NP, (F) NA, (G) MP, and (H) NS. Tip colors indicate the country of origin.
The PB2 segment distinguishes two groups among Uruguayan viruses detected in 2026 (SA-1a and SA-1b), which cluster with South American and North American low-pathogenic avian influenza lineages, respectively.
Across the remaining segments, Uruguayan viruses cluster within broader clades that also include viruses from Argentina and Brazil, consistent with a shared genomic background. Within these clades, a weak phylogenetic structure is observed, with sequences from SA-1a and SA-1b tending to form distinct subclades.
PB1 and PA cluster within South American low-pathogenic lineages (LP-SA), NP within North American lineages (LP-NA), and HA, NA, and MP within the Eurasian H5N1 clade 2.3.4.4b backbone. Branch lengths are proportional to the number of substitutions per site, and support values are shown at key nodes.

A (segment 1: PB-2)

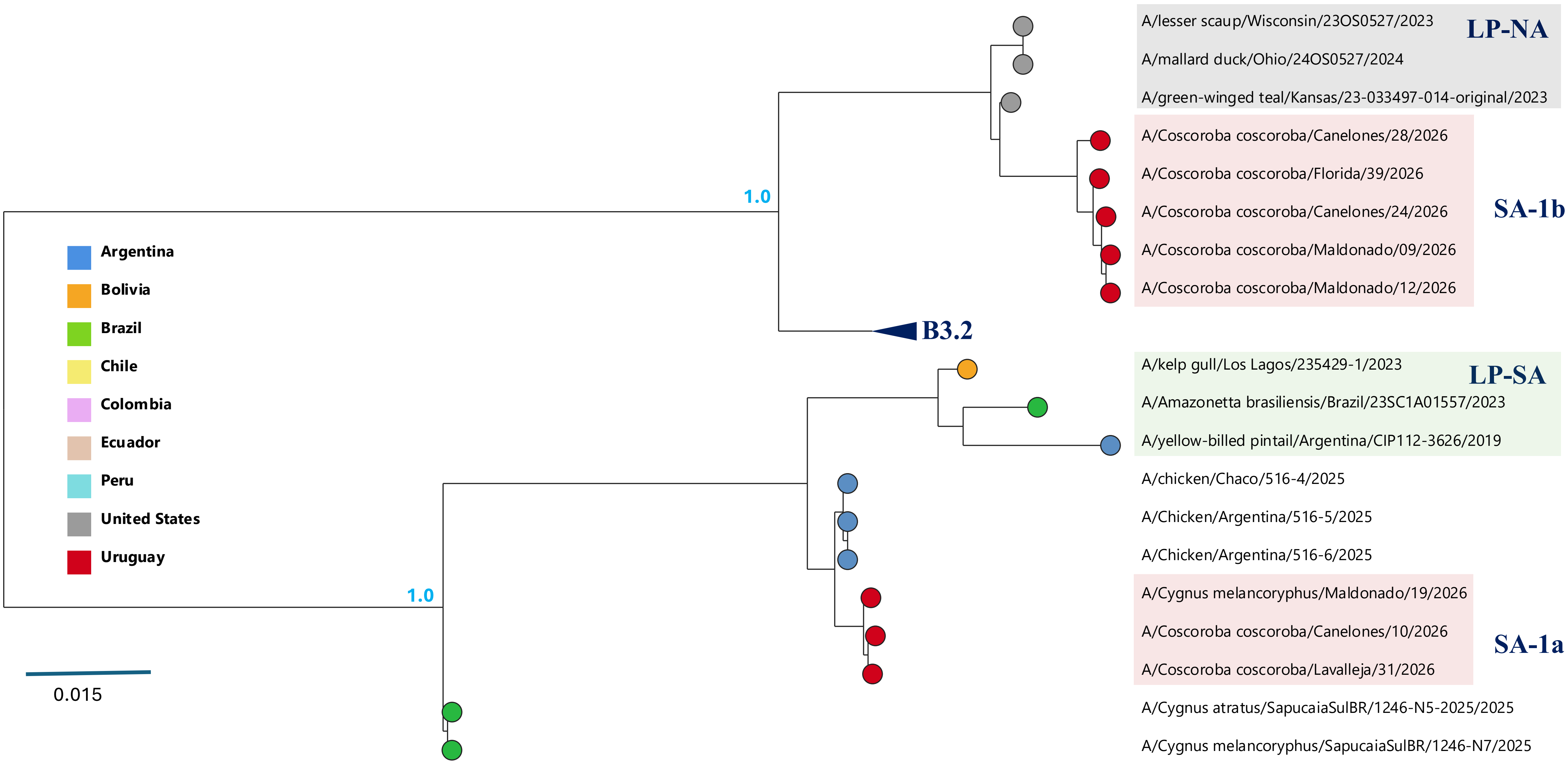

B (segment 2: PB1)

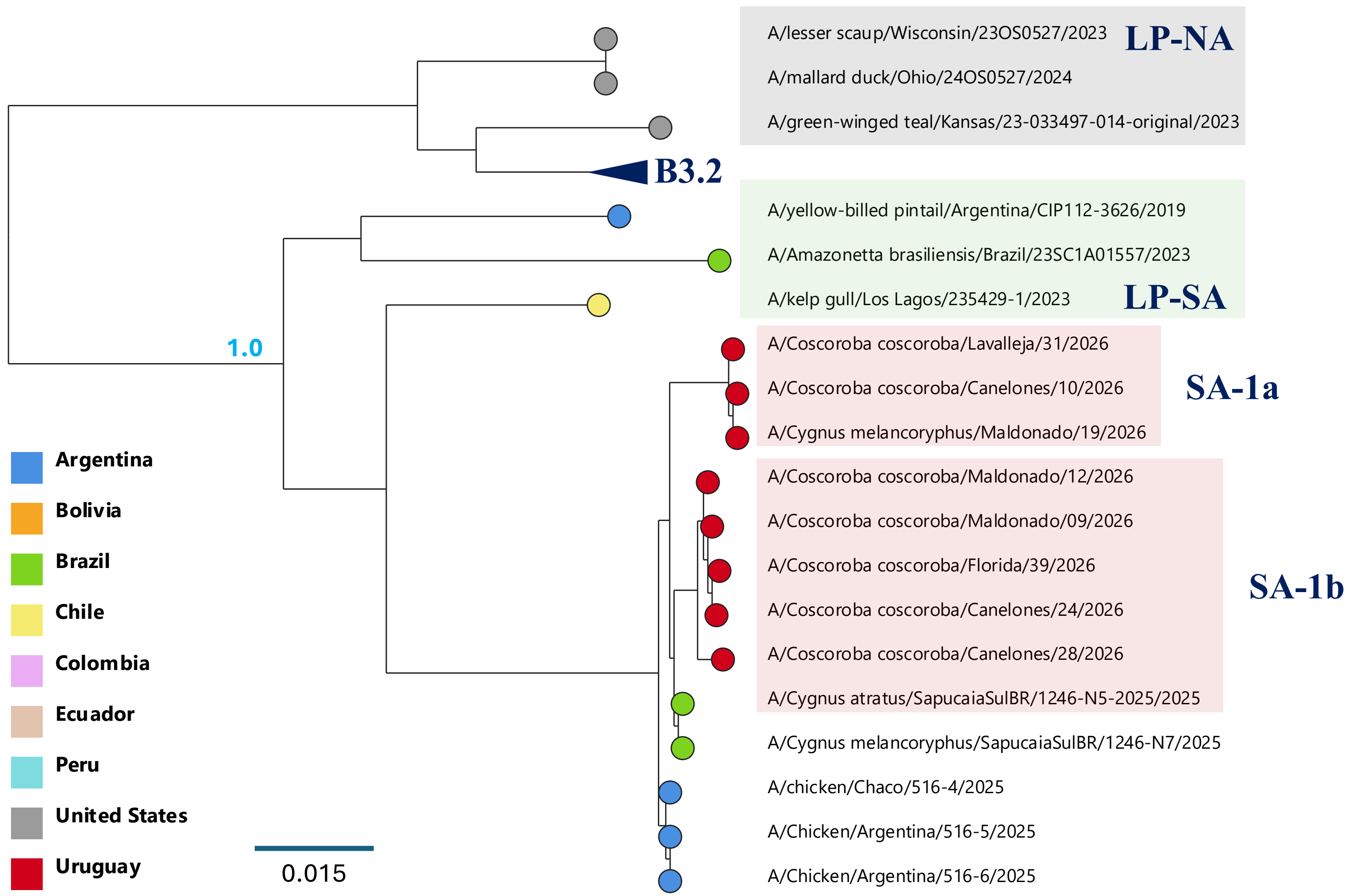

C (segment 3: PA)

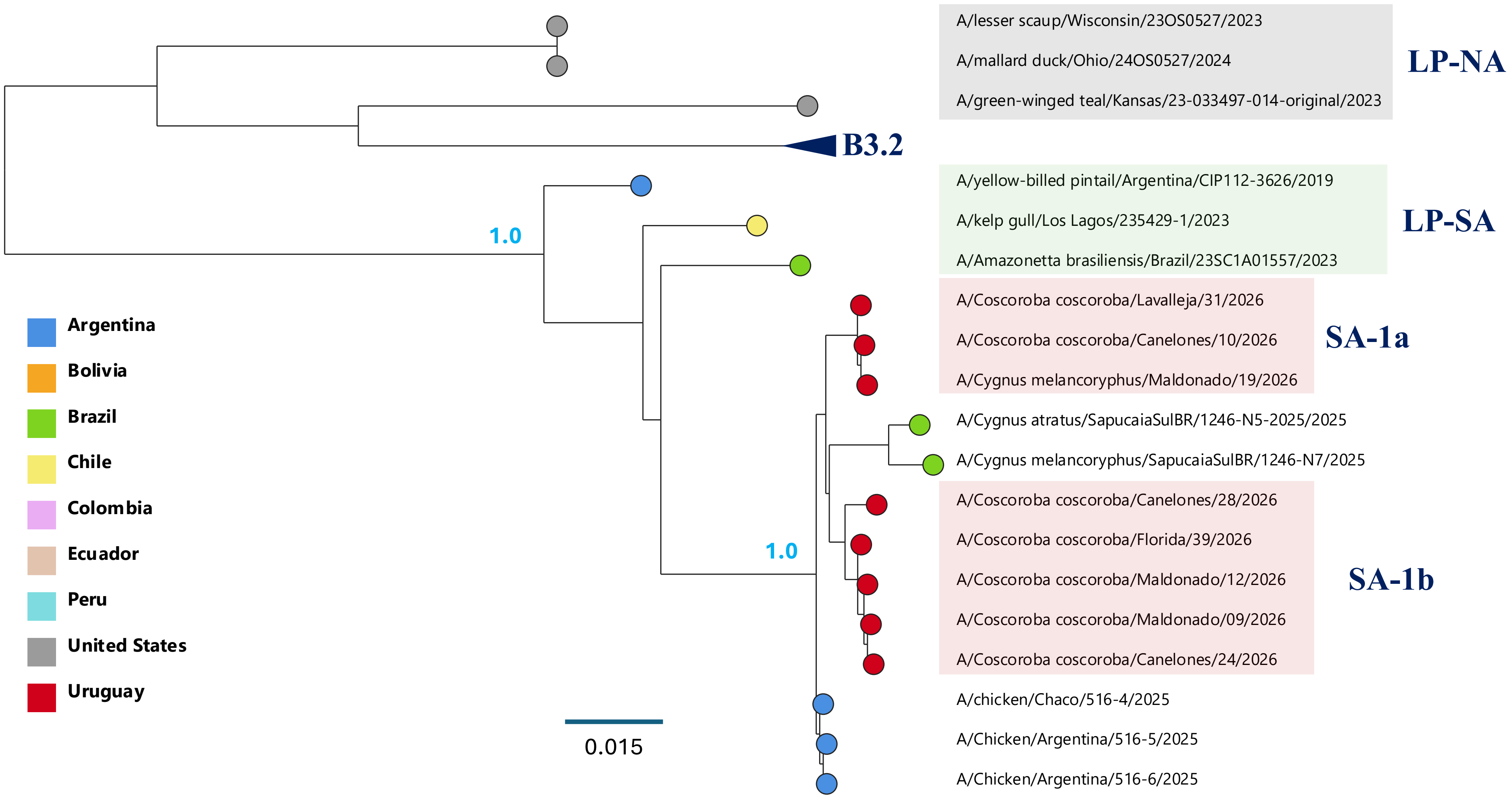

D (segment 4: HA)

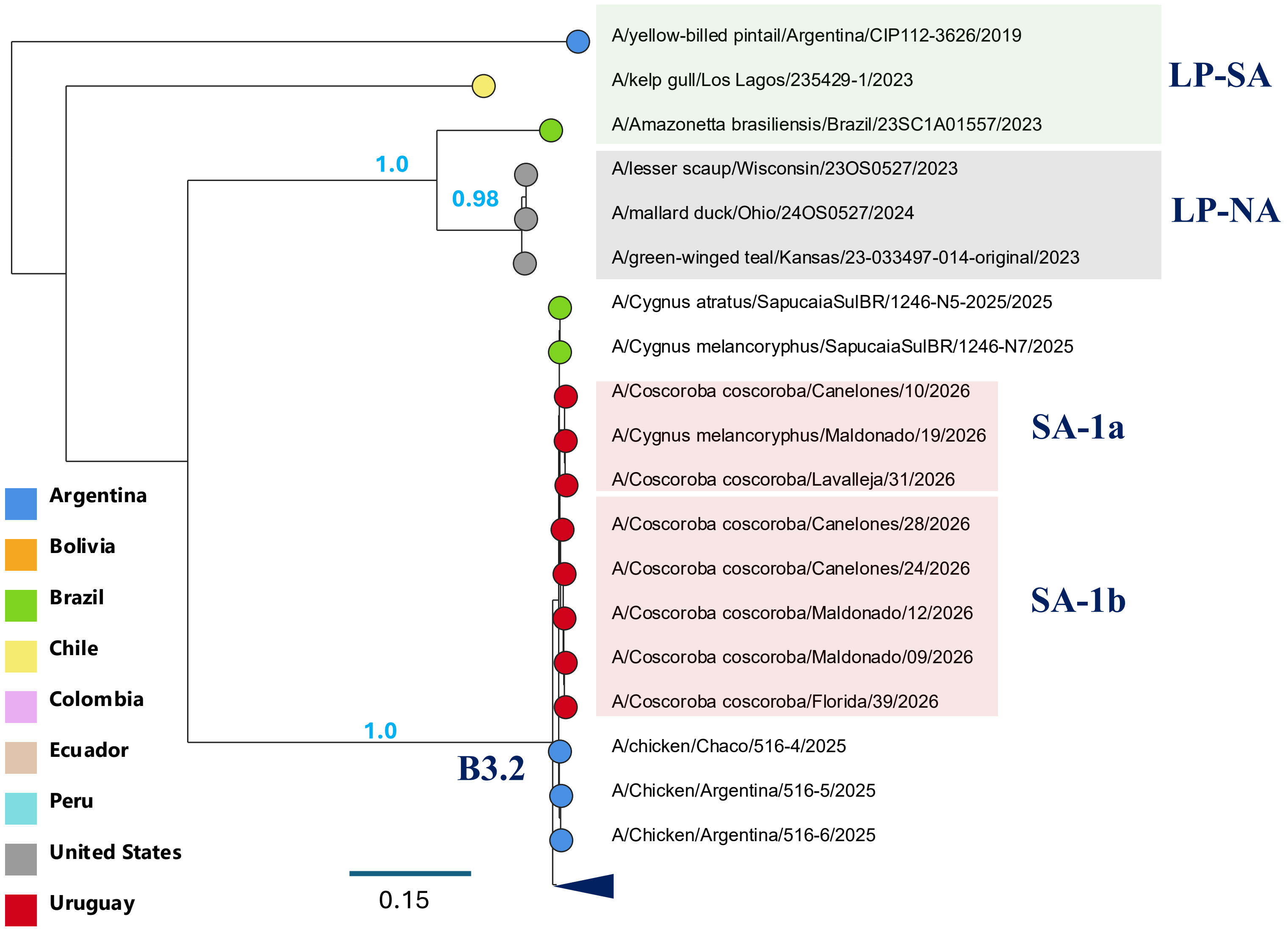

E (segment 5: NP)

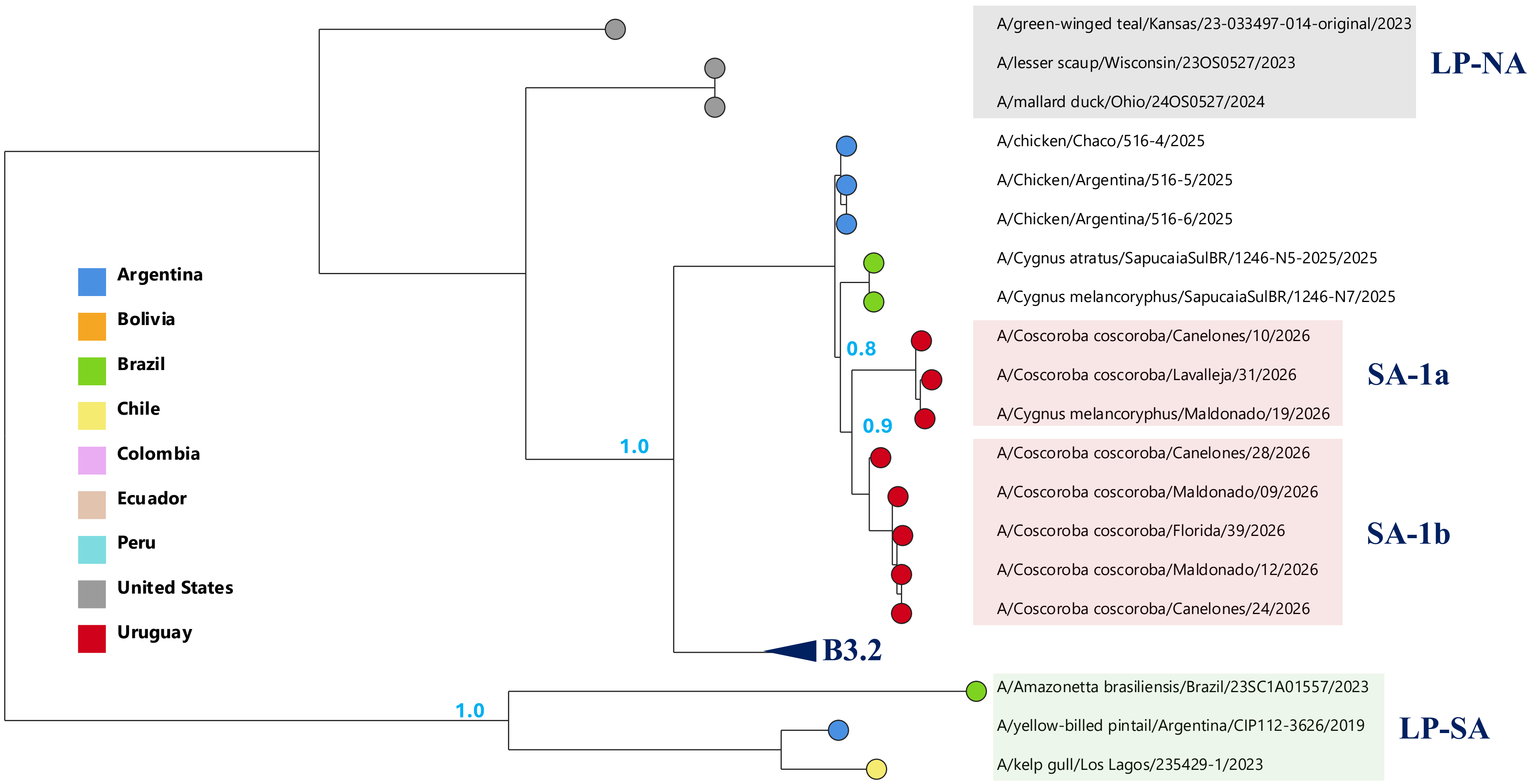

F (segment 6: NA)

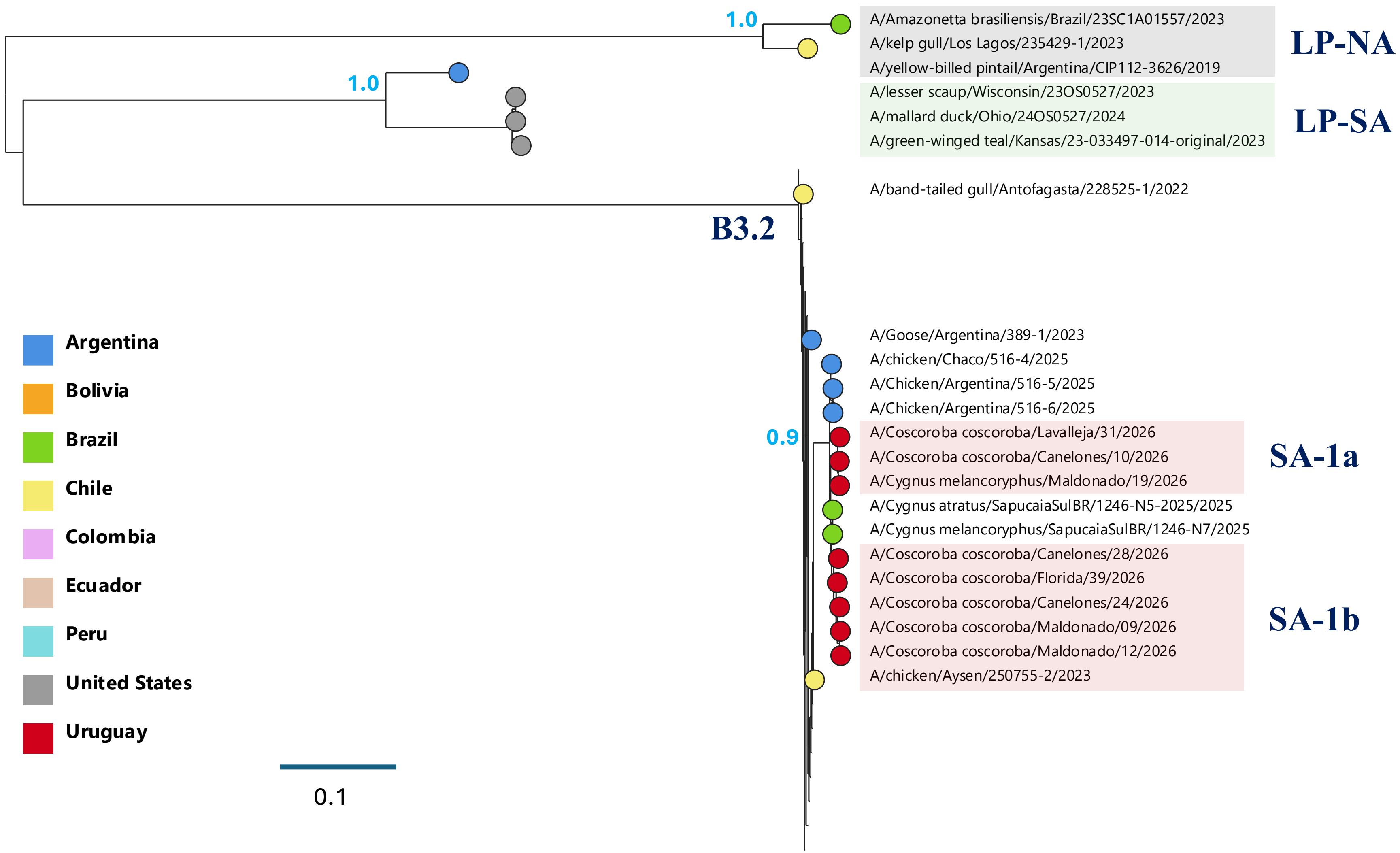

G (segment 7: MP)

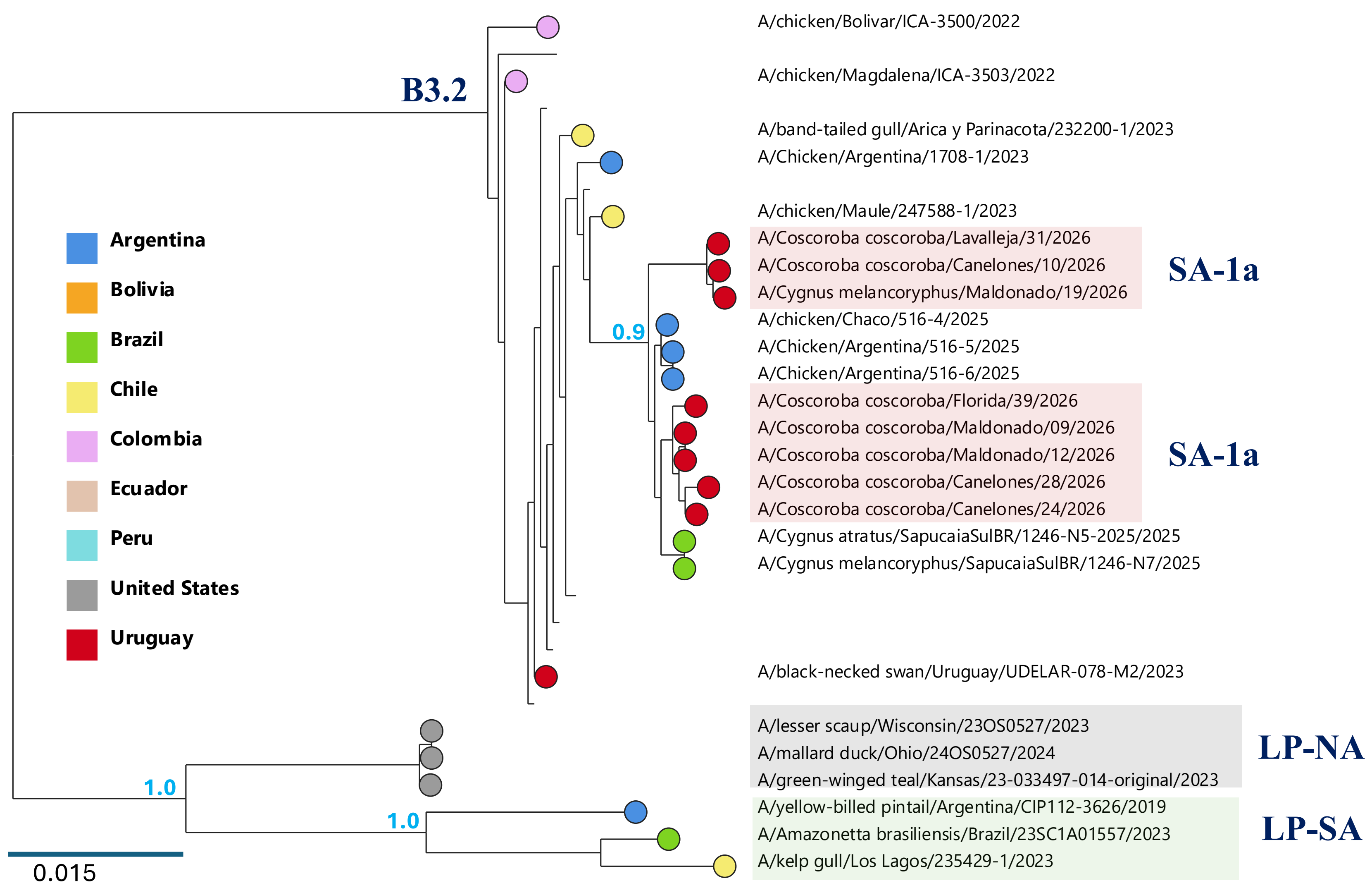

H (segment 8: NS)

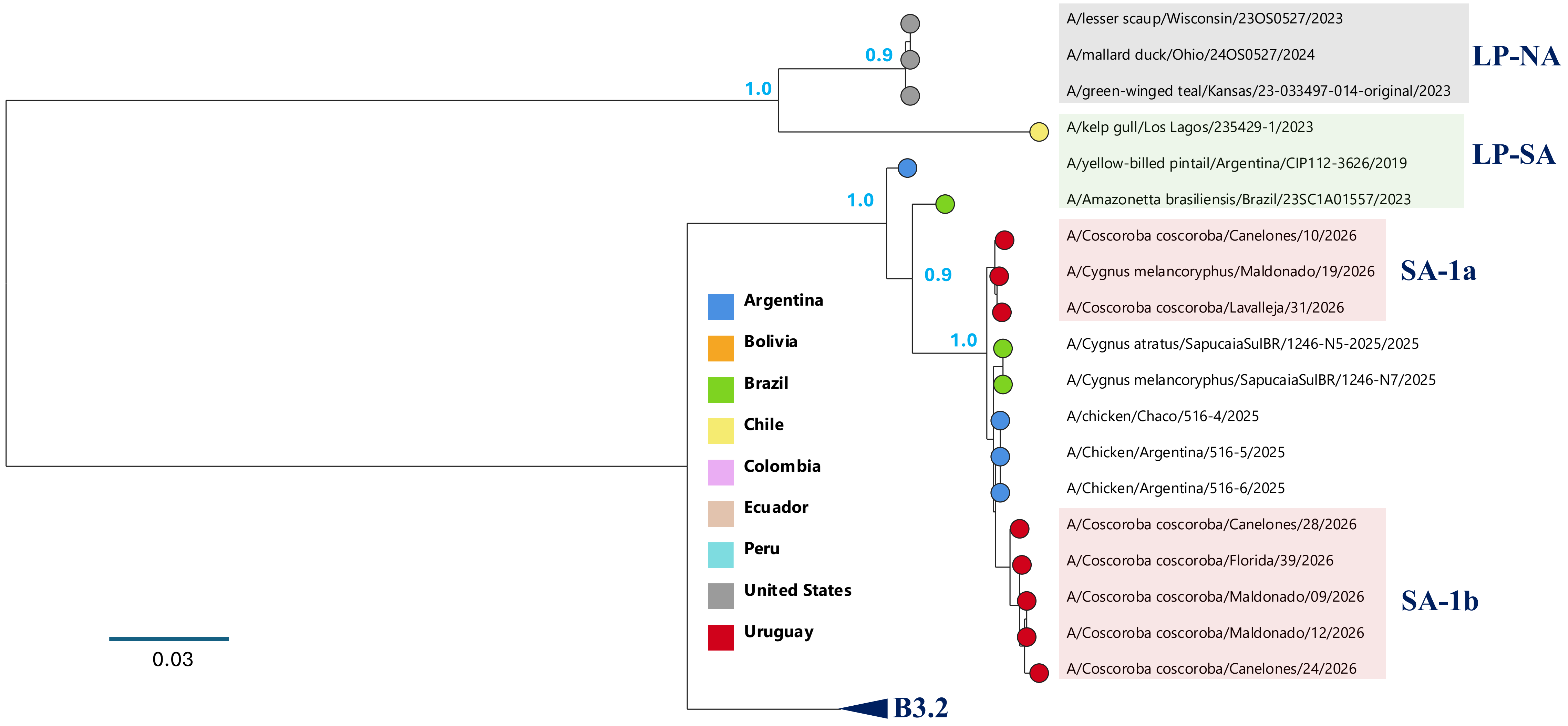

Supplement: Supplementary file 1 [file viruses-18-00558-s001.zip › Supplementary Figure S2.pdf]
